# The immunological function of CXCR2 in the liver during sepsis

**DOI:** 10.1186/s12950-022-00321-y

**Published:** 2022-11-30

**Authors:** Na Liu, Michael Bauer, Adrian T. Press

**Affiliations:** 1 Department of Anesthesiology and Intensive Care Medicine, Jena University Hospital, Am Klinikum 1, 07747 Jena, Germany; 2 Center for Sepsis Control and Care, Jena University Hospital, Am Klinikum 1, 07747 Jena, Germany; 3 Medical Faculty, Friedrich-Schiller University Jena, Kastanienstr. 1, 07747 Jena, Germany

**Keywords:** Sepsis, Liver, CXCR2, CXCL8, IL-8, Organ failure, Inflammation, Infection

## Abstract

**Background:**

The chemokine receptor CXCR2 and its ligands, especially CXCL8, are crucial mediators for the progression of liver inflammation and liver failure in sepsis. Neutrophils have the highest CXCR2 expression in mice and humans, and their activation via CXCL8 facilitates their migration to the inflamed liver for the clearance of the pathogens and, in turn, the inflammation.

**Main body:**

In sepsis, the inflammatory insult causes extensive neutrophil migration to the liver that overwhelms the immune response. To compensate for the strong receptor activation, CXCR2 desensitizes, incapacitating the immune cells to efficiently clear pathogens, causing further life-threatening liver damage and uncontrolled pathogen spread.

**Conclusion:**

CXCR2 function during infection strongly depends on the expressing cell type. It signals pro- and anti-inflammatory effects that may prompt novel cell-type-specific CXCR2-directed therapeutics.

**Supplementary Information:**

The online version contains supplementary material available at 10.1186/s12950-022-00321-y.

## Introduction

Sepsis is initiated through infections leading to a systemic dysregulated immune response syndrome resulting in an imbalance of pro- and anti-inflammatory responses. This significant damage to the host is clinically diagnosed as life-threatening organ failure [1]. 48.9 million people are diagnosed with sepsis yearly, with the latest estimate of 20% sepsis-related death in 2017. The liver represents a dominant integrator of pro- and anti-inflammatory signals during sepsis. Depending on the inflammatory stimuli, the liver-specific cells secrete inflammatory molecules modulating inflammation and adapting metabolically. These important mechanisms are at high risk of failure during sepsis [2]. Clinical observations highlight the importance of hepatic immune and metabolic signaling during infection. A pre-existing hepatic dysfunction makes the organism more vulnerable to infections, worsens sepsis outcomes, and is considered a decisive, independent risk factor for short- and long-term mortality [2–4].

Chemokine receptor signaling is a central coordinator of immunocyte trafficking during immune responses [5–7]. During this process, the chemokine CXC Ligand 8 (CXCL8) - Chemokine CXC receptor 2 (CXCR2, also known as IL-8RB, IL-8R2, IL-8Rβ) axis facilitates migration and secretion of inflammatory mediators critically in both the early and late phases of infection [8, 9]. The broad CXCR2 expression in immune and parenchymal cells facilitates various tissue-dependent signals, including migration, adhesion, proliferation, survival, and differentiation [10–12]. Leukocytes, mainly neutrophils and monocytes, constitutively express CXCR2. Moreover, fibroblasts, hepatocytes, and neurons display CXCR2 expression [13–15], where the CXCL8-CXCR2 axis stimulates cell death and regeneration [9, 16]. CXCR2 on hepatocytes regulates metabolic and immunological processes under basal conditions maintaining the liver’s tolerogenic environment [17]. In addition, the CXCL8-CXCR2 axis has shown significance, particularly in the liver during sepsis and the onset and progression of various liver diseases, such as alcohol or non-alcohol-related liver disease, hepatitis, cirrhosis, fibrosis, ischemia-reperfusion injury (I/R injury) [10–12]. Liver hepatocytes and resident immune cells monitor environmental changes and signals in the bloodstream. Once activated by antigens or insults, the release of CXCR2 ligands, especially CXCL8, from the liver triggers neutrophil recruitment. This results in the subsequent neutrophil-derived oxidative burst with cytotoxic granule release and the formation of neutrophil extracellular traps (NETs), eliminating pathogens and subsequent infection and inflammation control. Simultaneously, the chemokines and the triggered immune response exert significant hepatotoxicity, resulting in hepatocyte death and, eventually, liver failure [16, 18]. Consequently, the blockade of CXCR2 has immense potential to diminish the excessive production of inflammatory mediators and inhibit neutrophil-mediated liver damage [19–22]. Thus, the dosed interference with CXCR2 signaling may be a promising target for modulating the early and late dysregulated immune response that protects the liver from injury and failure during sepsis, associated with poor short, mid and long-term survival.

## The construction and conservation of CXCR2

CXCR2 is a G protein-coupled receptor (GPCR) assembled by seven transmembrane domains. The N-terminus of CXCR2 is located on the exterior front of the cell surface, facilitating ligand specificity. The C-terminus consists of heterotrimeric G proteins [23] that comprise three extracellular and three intracellular loops into the cytoplasm [24] essential for receptor signaling and activation-induced internalization. CXCR2 is highest expressed on neutrophils, monocytes, and lymphocytes (T cells, mast cells, and NK cells) participating in chemotaxis [25]. In contrast, in non-immune cells (hepatocytes [13, 26], fibroblasts [27], keratinocytes [28], adipocytes [29], neurons [30], epithelial [31], and endothelial cells [32]), CXCR2 is able to stimulate cell death [33], regeneration [34] and inflammatory responses [15, 35], including the expression of adhesion molecules, like platelet-endothelial cell adhesion molecule-1, in endothelial cells [36].

**Fig. 1:**
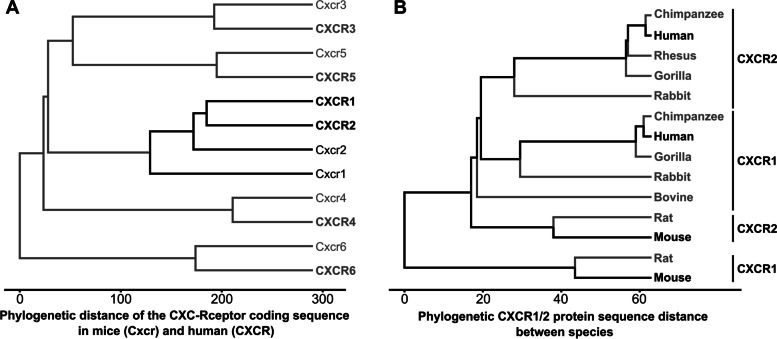
Conservation between the CXC receptor. **A** Phylogenetic distance of Chemokine CXC receptors (CXCRs) in mice and humans. Human (CXCR) and mouse (Cxcr) coding sequences are utilized to compute the phylogenetic relation and distance between the CXCR1-6. **B** Calculated phylogenetic distance of CXCR1 and CXCR2 protein between different animal species. Sequence data and the analysis parameters are provided in Supplementary Information 1

CXCR2 shares remarkable sequence similarity with CXCR1 (IL-8RA, IL-8R1, IL-8Rα), reaching a maximum of 77% over the membrane-spanning regions. However, despite this distinctive sequence similarity and conservation of CXCR2 and CXCR1 in humans (Fig. 1A), both receptors vary considerably in their ligand affinities and functions. Both receptors get activated by Glu-Leu-Arg containing (ELR+) chemokines. Diverging sequences in both receptors’ N- and C-terminal regions cause CXCL8, commonly known as IL-8, to interact with CXCR1 at a higher affinity than CXCR2, while CXCR2 interacts preferentially with all other ELR + chemokines (i.e., CXCL1-3, 5–7) [37]. The homologous CXCR1 and CXCR2 sequences from the gorilla, chimpanzee, rhesus, and orangutan were cloned and sequenced. Those studies found that the CXCR2 genes from four non-human primates are 95 to 99% identical to their human homolog. At the same time, rabbit CXCR2 has an 80% amino acid identity to the human, highlighting the essential functions of those chemokine receptors in the immune response across species. (Fig. 1B)

While the chemokine receptors share a great homology of structures between species, their respective ligands vary. CXCL8, also known as neutrophil-activating peptide-1 (NAP-1) or Interleukin 8 (IL-8), was the first recognized chemokine and is a pro-inflammatory mediator in humans [10–12]. In rodents, however, the core ligands are Cxcl1 (keratinocyte-derived chemokine, KC) and Cxcl2 (macrophage inflammatory protein 2, MIP-2) [38, 39]. Interestingly, murine Cxcl1 shares the highest sequence homology with human CXCL1, while mouse Cxcl is the functional homolog to human CXCL8 [40, 41]. CXCL8 elicits various biological processing via binding and activating its three central receptors, namely, CXCR1, CXCR2, and the Duffy antigen receptor for chemokines (DARCs) [42]. Despite lacking the CXCL8 gene, murine and rat neutrophils respond to hCXCL8 similarly to humans, mainly through Cxcr2. Cxcr2 in rodents plays a dominant role in the biological response of Cxcl1, while Cxcr1 in mice was newly identified to recognize human CXCL5 and CXCL8 [43, 44]. Different neutrophil chemoattractants bind the Cxcr2 receptors endogenously in rodents, taking over the CXCL8 function in humans [45]. Further, rodent Cxcr2 has an exceptionally high affinity to murine Cxcl1, Cxcl2, and Cxcl3 (Dendritic cell inflammatory protein-1, DCIP-1), thus binding the murine counterparts of human growth-related oncogenes (GROs) [46–48]. The activation of CXCR2 (for humans) or Cxcr2 (for mice) through CXCL8 (human) or Cxcl1 (mouse) both results in calcium influx and chemotaxis [7, 9, 46, 49]. The difference in homology and functions of Cxcr and Cxcl in humans and mice challenges the use of mouse models mimicking CXCL8-involved human diseases. In the past, however, recombinant human IL-8 (CXCL8) or homologous murine Cxcl1 had been found to have reasonable solutions with a good translational value. (Fig. 1) [45, 50].

## The activation of CXCR2

As the primary functional receptor for ELR + ligands, CXCR2 is emphasized as inflammation’s most essential and widely explored chemokine receptor. Once activated by CXCLs, CXCR2 dissociation with the G-protein induces the release of the Gβγ subunits from the Gα subunit. The dissociation causes downstream activation of phospholipase C (PLC, β-2 isoform), followed by calcium mobilization from the endoplasmic reticulum to cytosol and activation of protein kinase C [16]. Additional CXCR stimulation may result in the activation of various other signaling cascades, namely phosphatidylinositol-3 kinase (PI3K)/Akt, mitogen-activated protein kinase (MAPK)/p38 (but not JNK), Ras/Erk, and the Janus kinase (JAK2)/signal transducer and activator of transcription (STAT3) signaling associated with cytoskeletal remodeling and inflammation [8, 51–53]. For example, activated PI3Ks regulate neutrophil migration downstream [54], and MAPKs are involved in cell proliferation and survival [55].

**Fig. 2:**
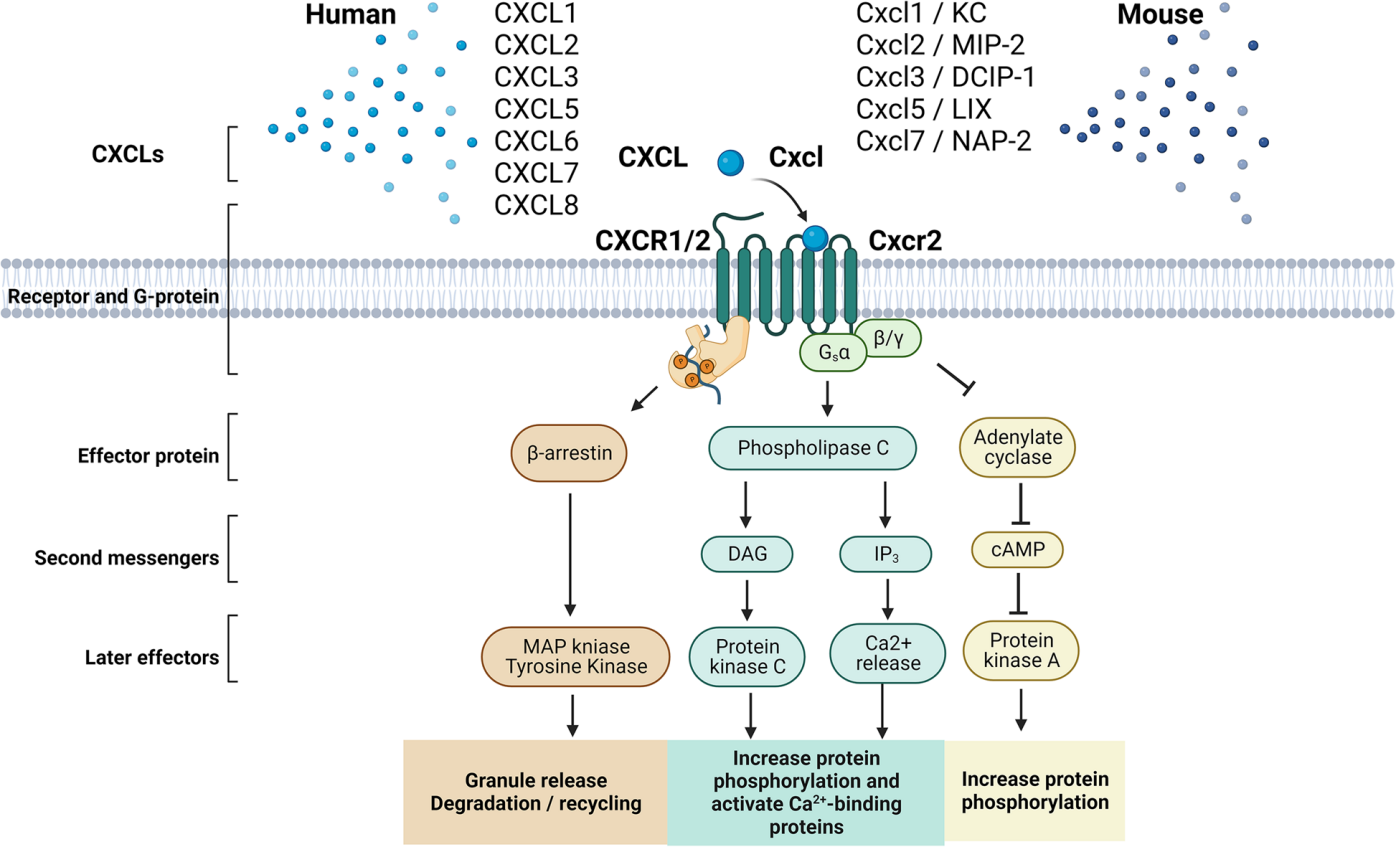
CXCLs-CXCR1/2 signaling cascades for humans and mice. Macrophages, monocytes, and endothelial cells release inflammatory chemokines. In humans, CXCL1-3, 5–8, and in mice, Cxcl-3, 5, 7, are released that bind to human CXCR1 and 2 or murine Cxcr2 in responsive cells (e.g., neutrophils, hepatocytes, and stellate cells). Once activated by CXCLs, the receptor dissociates with the G-protein with the release of the Gβγ subunits from the Gα subunit, which causes activation of phospholipase C (PLC, β-2 isoform) and subsequent calcium mobilization from the endoplasmic reticulum to cytosol and activation of protein kinase C, which lead to increased protein phosphorylation and calcium-binding. While for the Gβγ subunits, adenylate cyclase is inhibited, resulting in decreased cyclic AMP production and subsequent protein kinase A, with increased protein phosphorylation as well. In addition, β-arrestin1/2 regulates receptor internalization with MAP kinase and subsequently drives cell degranulation and receptor internalization for further degradation or recycling

CXCR2 signaling is further diverted by its ligand sensitivity. Thus, different ligands acting on CXCR2 elicit different cellular processes. Therefore, the ligands preferentially stabilize different active conformational dynamics of the receptor. This phenomenon is termed biased agonism [55] and is not only sensitive to its ligand but also different CXCL8 variants [56] and concentrations [57]. Ultimately, after the receptor activation, C-terminal phosphorylation recruits β-arrestin 1/2 that mediates synergistically with MAPK cell degranulation and receptor internalization for further degradation or recycling (Fig. 2) [51–53].

## The CXCR2 immunology in liver homeostasis and diseases

The liver is the largest solid organ in the body and is characterized by its metabolic, synthesis, detoxifying, and, importantly, immunological function [56]. The liver is enriched by unique, innate immune cells comprising KuCs. Many macrophages, natural killer cells, neutrophils, and monocytes build up an essential part of the host’s first defense line. Inflammatory signaling in the liver is dominated by an anti-inflammatory response, creating a tolerogenic environment where the daily flood of microbial compounds and microbes is eliminated without causing systemic signs of inflammation [57].

The majority of liver cells, constituting hepatocytes, liver sinusoidal endothelial cells (LSECs), KuCs, and hepatic stellate cells (HSCs), also function as antigen-presenting cells (APCs). They react to circulating antigens, microbial-associated molecular patterns (MAMPs), and damage-associated molecular patterns (DAMPs) from circulating immune cells and microorganisms. Those particles are eliminated, and anti-inflammatory factors are secreted, preventing a detrimental steady inflammatory state in the body. Thus, their immune surveillance is a powerful firewall against harmful and potentially dangerous particles that maintain local and systemic homeostasis [12, 58–60].

However, when danger signals overcome a threshold, inflammatory KuCs react with the initial antigens, microbiological components, cytokines, and adaptive immune cells, including specialized T cells and natural killer T cells that reside and patrol in the liver sinusoids to fight the source of the stimulus [12, 18, 59–64]. CXCR2 is expressed in various liver resident cells and may be involved in immune surveillance, supporting the overall tolerogenic environment. For example, CXCR2-expressing hepatocytes release cytokines and exosomes to maintain hepatocellular homeostasis independent of ligand binding [13, 65, 66]. This CXCR2-dependent network supports the maintenance of liver homeostasis in health and allows a quick immune response to react to danger signals (Table 1) [67, 68].

**Table 1:** Immune competent cells in the inflamed liver and their immunological function

Liver cells	Changes and functions	Secreted cytokines and other mediators	Ref
**Hepatocytes**	Present antigens; protein synthesis; energy metabolism; secrete and respond to immune proteins (e.g., acute phase proteins)	EGF, IGF-1, IL-1, 6, 8, 9, MCP-1, MCP-2, TNF-α, VEGF, NO, ROS	[59, 60, 69, 70]
**Liver sinusoidal endothelial cells**	Barrier formation; present antigens; endocytosis; produce and respond to immune mediators	HGF, IL-RA, IL-1, 6, 9, 18, 10, 33, TGF-β, TNF, NO	[59, 62, 69, 71–73]
**Dendritic cells**	Present antigens; phagocytosis; produce and respond immune mediators	IL-6, IL-10, IL-12, IL-15, IL-18, IL-21, TGF-β	[59, 63]
**Kupffer Cells**	Present antigens; endo- and phagocytosis; produce and respond to immune mediators	IL-1, IL-1RA, IL-6, IL-10, IL-12, IL-18, TNF- α, TGF, NO, CO	[60, 70, 74]
**Macrophages**	Migrate; secrete and respond to immune mediators; endocytosis; phagocytosis.	IL-6, IL-8, IL-10, TNF- α, VEGF, NO, ROS	[75, 76]
**Hepatic stellate cells**	Present antigens; liver fibrosis	TGF-β, IGF, IL-1, IL-6, IL-10, MCSF	[77, 78]
**Myeloid-derived suppressor cells**	Suppress T-cell activation; produce immunosuppressive mediators	IL-10, TGF- β, arginase	[8, 79]
**Neutrophils**	Chemotaxis; produce and respond to immune mediators; NET-formation	IL-4, IL-8, TNF- α, MPO, ROS	[73, 80, 81]
**Natural Killer cells**	Cytotoxicity; produce and respond to immune mediators	IFN-γ, IL-6, TNF	[82, 83]
**T cells**	Activation; differentiation; proliferation; effective molecules production; cytotoxicity	IL-2, IL-4, IL-6, IL-10, IFN- γ	[59, 69, 84, 85]
**B cells**	Activation; differentiation; proliferation; effective molecules production; secrete antibody	IL-6, IL-10, IL-12, TNF-α, GM-CSF	[63, 86]

*EGF* Endothelial growth factor, *MCP* Monocyte chemotactic protein, *VEGF* Vascular endothelial growth factor, *IL-1RA* IL-1 receptor antagonist, *IGF* Insulin-like growth factor, *MCSF* Macrophage colony-stimulating factor, *TNF-α* Tumor necrosis factor-α, *NET* Neutrophils extracellular trap, *MPO* Myeloperoxidase, *NO* Nitric oxide, *ROS* Reactive oxygen species, *GM-CSF* Granulocyte-macrophage colony-stimulating factor

This inflammatory response is a predominant contributor to the pathogenesis of liver diseases, and the CXCL8-CXCR2 axis is essential for liver inflammation via recruiting neutrophils at the site of infection. CXCL8 mediates the infiltration and proliferation of immune cells, predominantly neutrophils, in the liver [10, 21, 87]. LSECs and KuCs secrete CXCL8 in the liver [88] in response to alarmins and infection [15, 20, 89] attract immune cells so as to clear pathogens, cellular debris, alarmins, and metabolic waste restoring liver function [59, 90–92]. Through the CXCR1/2-CXCL8-axis, attracted neutrophils then produce antimicrobial mediators, like ROS and proteases, or undergo NETosis to localize the infection that goes along with liver cell injury [58]. Pharmacological CXCR1/2 inhibition and compensatory CXCR1/2 down-regulation can restrict hepatotoxicity by reducing the neutrophil migration into the tissue [85]. In contrast, liver cells dying through non-apoptotic pathways release pro-inflammatory DAMPs, further aggravating hepatitis and injury [58, 93]. Meanwhile, immune cell recruitment results in the aggregation of collagen and fibrosis, which worsens liver inflammation [93, 94]. In chronic infections, excessive inflammation results in immune paralysis and an abnormal loss of hepatocytes. Both mechanisms accelerate liver damage in situations of an ischemia-reperfusion injury, obesity and non-alcoholic fatty liver disorders, alcoholic hepatitis, and infection, ultimately resulting in irreversible liver damage, cirrhosis, and eventual carcinogenesis [18, 61, 95–97].

## The CXCL8-CXCR2 axis in the liver

The CXCL8 chemokine family, mainly responsible for inducing and maintaining the inflammatory state, is known for neutrophil activation and migration into the inflamed tissue, or neutrophil-mediated tissue injury, and plays an essential role in liver diseases [9]. The CXCL8-CXCR2 axis mediates communication among hepatocytes, HSCs, KuCs, and LSECs, with other liver residents and circulating immune cells [98]. Different chemokines targeting CXCR2 on the responsive cells stimulate the trafficking of immune cells to sites of liver inflammation or injury. For example, CXCL8 released from hepatocytes and LSECs upon infection causes chemotaxis of neutrophils and monocytes, changes endothelial cell permeability via cytoskeletal reorganization (Table 2) [5–7, 9, 12, 37, 58, 99].

**Table 2:** CXC chemokines and their receptors in the liver

Systematic name (mouse, human)	Name (mouse)	Name (human)	Chemotaxis	Receptors (mouse, human)	Expressed cells	CXCR2 Affinity (EC50, nmol/L)
**CXCL1**	KC	GRO-α, GRO1, MGSA-α, NAP-3	Neu, LSEC, Bas	CXCR1, CXCR2	Neu, Mon, Eos, Epi, LSECs, T cells	5
**CXCL2**	MIP-2	GRO-β, GRO2, MGSA-β, MIP-α	Neu, LSEC, Bas	CXCR2	Neu, T cells, Mac	4
**CXCL3**	DCIP-1	GRO-γ, GRO3, MGSSA-γ, MIP-β	Neu, LSEC, Bas	CXCR2	T cells, LSECs, Mac	1
**CXCL5**	LIX	ENA-78	Neu, LSEC	CXCR2	Eos, Epi, LSECs, Mac	11
**CXCL6**	N/A	GCP-2	Neu, LSEC	CXCR1, CXCR2	LSECs, Mac, Neu	N/A
**CXCL7**	NAP-2	PPBP, NAP-2	Neu, LSEC, Bas	CXCR1, CXCR2	Mon, T cells, DCs, Mac	7
**CXCL8**	N/A	IL-8, NAP-1	Neu, LSEC, Bas, Mon	CXCR1, CXCR2	Mon, T cells, Mac, Epi, Hepa, LSECs, Neu	4

*GRO-α/β/γ* Growth-related oncogene, *IL-8* Interleukin-8, *ENA-78* Epithelial cell-derived neutrophil-activating protein-78, *PPBP* Pro-platelet basic protein, *NAP-2* Neutrophil-activating peptide-2, *GCP* Granulocyte chemotactic protein 2, *KC* Keratinocyte-derived chemokine, *MIP-2* Macrophage inflammatory protein-2, *LIX* Lipopolysaccharide-induced CXC human chemokine, *DCIP-1* Dendritic cell inflammatory protein-1, *Neu* Neutrophils, *LSECs* Liver sinusoidal endothelial cells, *Bas* Basophils, *Eos* Eosinophils, *Mon* Monocytes, *Mac* Macrophages, *Hepa* Hepatocytes, *HSCs* Hepatic stellate cells, *Epi* Epithelial cells, *DCs* Dendritic cells, *EC50* half maximal effective concentration

Once neutrophils reach the tissue, CXCR2 activation induces the release of granule enzymes, ROS, and NETosis to eliminate pathogens [79]. The same CXCL8-CXCR2 axis regulates lymphocyte trafficking to inflamed body regions [100]. Upregulation of CXCL8 expression in those cells, along with the infiltration and accumulation of immune cells during pathological conditions, correlates with chronic and dysregulated inflammation in the liver [101, 102].

LSECs constitute the wall of the hepatic sinusoid, connecting blood and hepatocytes via their fenestrations called sieve plates. They also activate neutrophils and facilitate their transmigration into the parenchyma. Through the neutrophil-mediated disruption of the endothelial barrier during chemotaxis, LSECs themselves suffer injury. In sepsis, chemotaxis is an uncontrolled and overshooting event that destroys the endothelial barrier, significantly contributing to liver inflammation and injury [73, 76]. Besides, CXCL8 secreted by cholangiocytes activates HSCs. Here, CXCR2 signaling then induces their differentiation into pro-fibrotic myofibroblasts [88], which contribute to the collagen and extracellular matrix (ECM) deposition. Well-dosed ECM deposition supports the regeneration of the liver architecture; however, if the inflammation turns chronically, aggravated ECM deposition by activated HSC and myofibroblasts becomes a hallmark of liver fibrosis [103]. In the course of infection, inflammation also enhances CXCR2 expression on hepatocytes and cholangiocytes, resulting in their proliferation and angiogenesis, which are vital for liver regeneration [19, 74, 91]. A second important mechanism occurs after prolonged or repeated activation of CXCR2 on chemokine-targeted cells, like neutrophils, that respond to this stimulus with receptor desensitization and internalization [100, 104, 105]. This desensitization and pharmacological antagonization of CXCR2 significantly decreased neutrophil migration to the injury sites and increased the local pathogen burden despite the local ligand concentration [100]. Since neutrophils injure hepatocytes by releasing ROS, NETs, and proteinases, desensitization of CXCR2 may be considered protective in the liver. However, CXCR2 signaling in hepatocytes can result in their repair or death depending on local ligand concentrations and independent of the accumulation of activated, and thereby toxic, neutrophils with CXCR2 [15, 62, 101, 102]. Consequently, the desensitization may not only result in the protection of parenchymal cells from neutrophil-mediated injury but also a chronic spread of pathogens and further dysregulation of inflammatory signaling. Additionally, inhibition of regenerative processes through CXCR2 desensitization in non-immune cells may further promote chronic liver diseases.

Observations in various clinical and preclinical settings uncover CXCR2’s paradoxical role in regulating responsive cells, especially neutrophils, based on their ability to produce inflammatory mediators for host defense counteracting PAMPs and DAMPs potential hepatotoxicity. Based on CXCR2’s pivotal role in liver inflammation, many efforts have been taken to establish a CXCR2-related treatment for liver diseases, including alcohol-associated liver disease (ALD), non-alcoholic fatty liver disease (NAFLD), viral hepatitis, ischemia & reperfusion injury (I/R injury), cirrhosis, and fibrosis. Overall, liver function suffers from CXCLs-CXCR2 signaling during inflammation (Table 3) [13, 15]. Therefore, targeting CXCR2 to inhibit neutrophil infiltration and activation, thereby protecting liver resident non-immune cells, raises a potential therapeutic target to support the host response to infection, reducing hepatotoxicity [19, 73].

**Table 3:** Role of CXCLs-CXCR2 in human liver diseases

Diseases	Cytokines and Chemokines	Proposed role in disease	Function in animal models	Reference
ALD	CXCL1,4,5,6,8, TNF-α	Neutrophil chemoattractant; increased expression were biomarkers for poor prognosis.	Antibodies neutralizing CXCLs or genetic deletion CXCR2 alleviated inflammation.	[98, 106]
NAFLD	CXCL8, IL-1, 6, 18, MCP-1, TNF-α	Immune cell recruitment, metabolic disorder, oxidative stress, and increased serum CXCL8 predicted the severity of hepatic fibrosis.	Antibody-mediated neutrophil depletion suppressed steatohepatitis and avoided tissue damage.	[61, 96, 107, 108]
Cirrhosis Fibrosis	PDGF, TGF-β, CCL2, 5, CXCL8,16, IL-4, 6,10, 13	CXCR2-mediated intracellular calcium mobilization and further neutrophil trafficking; biomarkers of cirrhosis progression; uncontrolled neutrophilic accumulation.	CXCR2 antagonist on neutrophil dysregulation and pro-inflammation states to prevent further cirrhosis.	[87, 109, 110]
Hepatitis B and C	CXCL8, IL-1, 6, 10, 18, TNF-α	Increased IL-8 accumulated neutrophils to the liver; decreased CXCR2 expression correlates with disease severity.	Inhibitors on IL-8 or CXCR2 downregulated inflammation response and alleviated hepatitis.	[20, 71, 111]
I/R injury	CXCL8, IL-1, 6, 11, 12, 13, 18, TNF- α	Neutrophil recruitment & activation; ligands production directly related to the duration of reperfusion; angiogenesis.	Blockade of CXCLs or CXCR2 and Cxcr2^−/−^ mice decreased local and systemic inflammation and promoted liver proliferation.	[13, 15, 112]
ACLF	CXCL8, ROS, IL-6, 17, 23, CCL-20, GM-CSF	Neutrophil chemotaxis to the site of inflammation/injury with high CXCR1/2 expression mediates the hepatic immune response.	Cxcr1/2 antagonist alleviated the production of inflammatory mediators and reduced cell death.	[20, 111, 113]
Sepsis	CXCL1, 2, 5, 8, TNF- α, ROS, iNOS, NETs	Neutrophil migration and its overstimulation led to CXCR2 internalization, microbial dissemination, uncontrolled systemic inflammation, and host death; the detection of CXCL8 predicted severity and evolution to organ failure.	Signaling prevents neutrophils’ failure to migrate to restore the sufficient levels of CXCR2 on their surface, including blockade or inhibition of PI3Kγ, and administration of IL-33 leads to better control of systemic inflammation and decreasing mortality.	[105, 114–120]

*ALD* Alcohol-associated liver disease, *NAFLD* Non-alcoholic fatty liver disease, *I/R injury* Ischemia & reperfusion injury, *ACLF* Acute-on-chronic liver failure

## CXCR2 in the liver during sepsis

Infection and inflammation are critical clinical manifestations of sepsis. The chemotaxis of neutrophils via CXCR2 from the circulating blood to the infection regions plays a vital role in sepsis [121]. Once neutrophils have found and recognized an invading pathogen, their phagocytosis and pathogen clearance abilities limit the infection [122]. Neutrophils are the most abundant immune cells in the periphery and have a relatively short life span. Neutrophils descend from granulocyte-monocyte progenitor (GMP) cells that differentiate into a neutrophil precursor population, further developing into immature and mature neutrophils. Already during the developmental process, CXCR2 maintains the neutrophil’s homeostasis [97, 123]. During neutrophil maturation in development and adults, CXCR2 upregulation and downregulation of its counter receptor CXCR4 promote mobilization of neutrophils from the bone marrow to the peripheral blood [124]. In addition, G-CSF signaling supports the proliferation and differentiation of GMP cells but cooperates with CXCL8-CXCR2 to release circulating neutrophils [125]. Through those processes, mature and immature neutrophils, namely myeloid-derived suppressor cells (MDSCs), accumulate in the peripheral blood and inflamed organs, like the liver in sepsis. MDSCs can suppress T cells mediated immune signaling vital for a targeted, adaptive immune response in sepsis [126–131].

CXCL8 is secreted from innate immune cells, including circulating neutrophils, monocytes, hepatocytes, liver resident LSECs, HSCs, and KuCs under acute and chronic inflammatory conditions. CXCL8 bound to CXCR2 mediates the migration of responsive cells and has various biological functions in eliminating pathogens and disease-related processes, like liver injury, fibrosis, and angiogenesis [9, 14, 52, 87, 98, 103]. CXCL8 expression in sepsis is profoundly increased and associated with sepsis’s progression and prognosis [118, 119, 132, 133]. This chemokine storm impairs all phases of neutrophil trafficking by stimulating CXCR2 internalization. Those phases include mobilization and release from the bone marrow, migration and rolling, adherence, and transmigration [114, 122, 134]. The patterns of dysfunctional neutrophils that lost their CXCR2 occur in septic patients, accompanied by suppressed CXCL8-induced chemotaxis ability of those cells [116, 135, 136]. This state is perilous as it accelerates pathogen spread and the uncontrolled release of cytokines. Thus, restoring the expression of CXCR2 on neutrophils might be potential sepsis therapy [137]. The expression of CXCR2 on neutrophils is regulated context-dependent, TNF-α, Nitric oxide, TLR2-, or TLR4-agonists in high concentrations downregulate CXCR2, similarly to persisting high CXCL8 levels [138–142]. DARC, as a substitute chemokine receptor for CXCR2, has high homology of CXCR2 with high affinity to ligands but without an actual ligand-related immune response and thus acts as decoy receptors limiting CXCR2 signaling [143–146].

The downregulation mechanisms of CXCR2 are diverse and tightly regulate its function during inflammation. On the molecular level, the inhibition of CXCR2 at the cell surface is closely connected with its internalization by endocytosis stimulated through β-arrestin 1/2 signaling [52, 141]. The expression of surface CXCR2 on neutrophils is widely considered a sepsis-specific biomarker that correlates to sepsis’s clinical severity and mortality [140, 147]. CXCR2 in sepsis is globally downregulated in bone marrow and neutrophils. CXCR2 downregulation in sepsis depends on its phosphorylation by the G protein-coupled receptor kinase-2 (GRK2) and the upregulation of a serin-threonine protein kinase [28, 123, 148]. The counteracting mechanism of CXCR2 internalization has also been identified to overcome sepsis-related receptor suppression [28, 123, 149]. Inducible nitric oxide synthase (iNOS) attenuates CXCR2 internalization and restores its function in sepsis [115]. At the same time, 2-deoxyglucose (2-DG), a glycolytic inhibitor for GRK2, reverses the impairments and results in CXCR2 expression on the surface of circulating neutrophils, increased migration, and their chemotaxis, respectively [150]. The down-regulation of neutrophil CXCR2 and inhibition of NET releases via phospholipase D2, a phosphotyrosine protein involved in the signaling of GRK2 and CXCR2, significantly enhances bactericidal activity [151]. NETs are extracellular scaffolds generated from neutrophils after CXCR2 activation during infection. NETs facilitate bacterial clearance via physically trapping microorganisms but implicate liver injury, alleviated by inhibiting NETs [105, 121, 148, 151]. NET release aggravates sepsis’s coagulation disturbance and organ failure [152–154]. However, despite some protective mechanisms, systemic CXCR2 inhibition ultimately aggravates organ damage and increases mortality in murine sepsis models [155]. Upregulation of CXCR2 via inhibiting p53-induced CXCR2 internalization improves sepsis prognosis for mice [156]. In contrast, activating CXCR2 via the extracellular matrix degradation product acetylates Pro-Gly-Pro protected mice from severe sepsis [148, 149, 155].

**Fig. 3:**
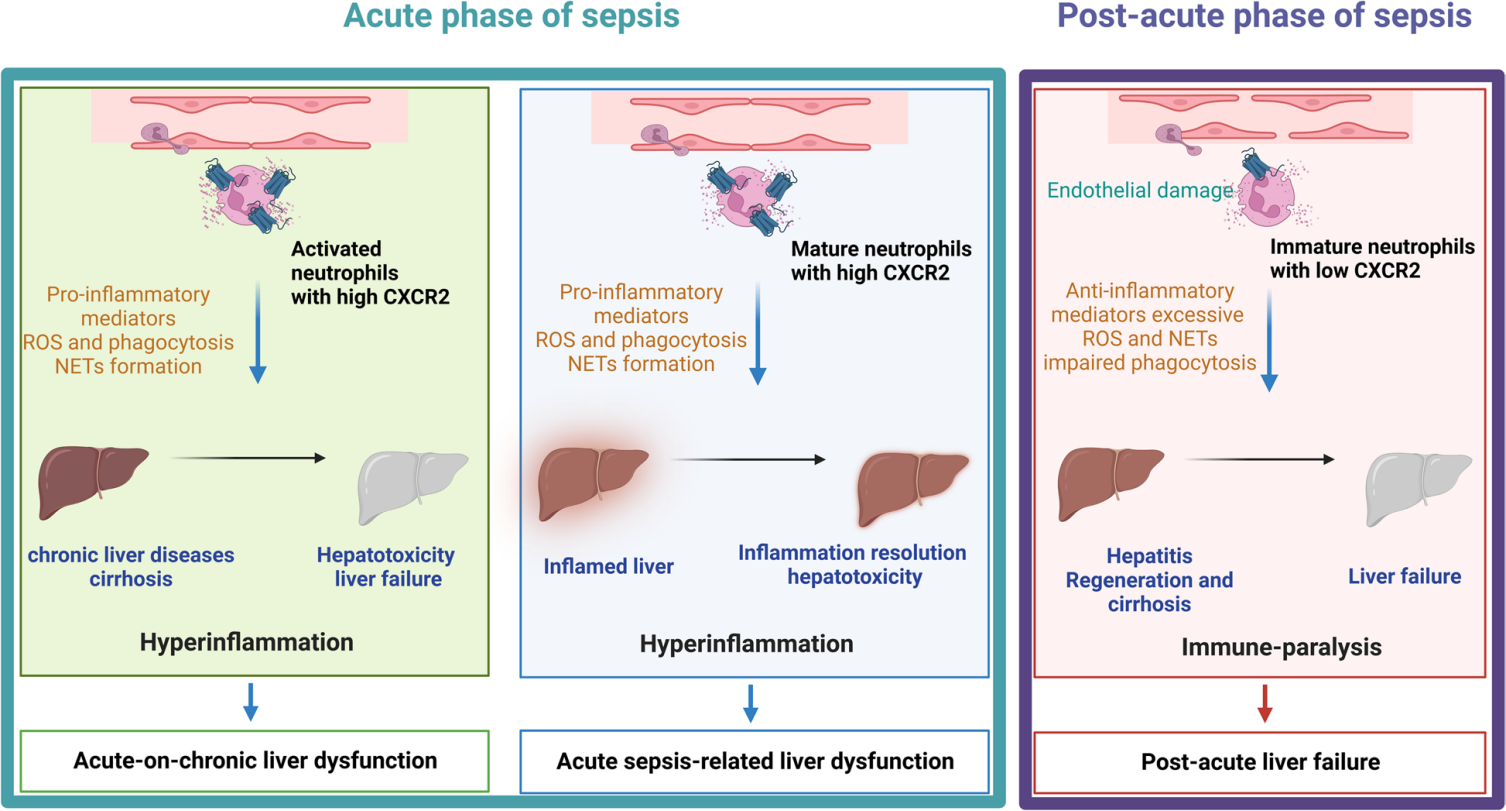
CXCR2 regulates neutrophils in the inflamed liver at acute and post-acute phases of sepsis. For the acute phase of sepsis, mature circulating neutrophils with high expression of CXCR2 activate and migrate from the blood flow to the liver via chemoattractant CXCLs, especially for IL-8 released from LSECs, KuCs, HSCs, and hepatocytes. Activated neutrophils display a wide range of effector mechanisms to counteract pathogens, which include the secretion of pro-inflammation mediators, ROS, phagocytosis, and NETs while damaging liver parenchymal cells as well. However, in the post-acute phase of sepsis, the endothelial barrier damage leads to immature neutrophils with lower expression of CXCR2 trafficking to the liver, with dysfunction of neutrophils, including migration, secretion of inflammatory mediators, and secretion and phagocytosis for pathogens. In addition, impaired neutrophils have suppressive immunity for other immune cells and excessive injury for the hepatocytes via ROS and NETs, which drive liver failure in sepsis. ROS, reactive oxygen species; NETs, neutrophils extracellular traps

Based on the distinct stage of sepsis, CXCR2 expressing neutrophils have various functions throughout the diseases. For the acute stage of sepsis, mature circulating neutrophils in the blood expressing high CXCR2 levels migrate from the bloodstream to the liver via chemoattractant (CXCL) gradients. Especially CXCL8 released from LSECs, KuCs, HSCs, and hepatocytes attract neutrophils potently. Activated neutrophils display a wide range of effector mechanisms to counteract pathogens, which include the secretion of pro-inflammation mediators, ROS, phagocytosis, and NETs. However, while those mechanisms aim to eradicate the infection, they cause significant liver cell injury if not tightly regulated. The aberrant accumulation of neutrophils in the liver and its subsequent immune response result in hyper-inflammation and hepatocyte death. Hence, it fails to control the immediate local and systemic inflammation [22, 102]. In contrast, severe chronic stages of sepsis show endothelial barrier damage leading to immature neutrophils with lower expression of CXCR2 entering the bloodstream. Their inability to traffic to the liver, to secrete inflammatory mediators, and to phagocytose pathogens impairs and gives them an immuno-suppressive function to other immune cells while causing excessive injury for the hepatocytes through an uncontrollable generation of ROS and NETs, driving sepsis liver failure (Fig. 2) [20, 120]. Numerous efforts focused on reversing CXCR2 defects and exploring the exact mechanisms of CXCR2-mediated neutrophil chemotaxis. For example, the blockade of PI3K restored CXCR2 surface levels on neutrophils via inhibition of GRK2 in a septic mouse model and translated into a better prognosis of sepsis. Similar observations with the inhibition of IL-33 elucidate CXCR2 as a promising target in sepsis therapy (Table 3 and Fig. 3) [114, 115, 117, 150, 156].

## CXCR2 is a potential therapeutic target for liver diseases

The CXCL8-CXCR2 axis is a promising biomarker for liver diseases. CXCL8-CXCR2 signaling is a marker for diagnosing Hepatitis B Virus infection and liver failure. Similarly, CXCR2 signaling acts as a pharmacological target in these diseases due to its intensive association with progression and prognosis [22, 71, 87, 111, 152]. Current treatments target the receptor and its ligands for neutrophil chemotaxis, mainly focusing on CXCR2 [20, 85]. Reparixin, a small molecular CXCR1/2 inhibitor, shows excellent tolerance and safety in first clinical phase I and II trials for I/R injury, typical in liver transplantation and early liver transplantation allograft dysfunction [13]. Blocking CXCR1/2 with cell-penetrating peptides called pepducins might increase survival and reverse hepatic inflammation and steatosis. However, CXCR1/2 inhibition does not entirely abolish neutrophil chemotaxis into tissues induced by other neutrophil chemoattractants [106]. In addition, CXCR1/2 blockade restrains systemic inflammation in mice with peritonitis and liver failure [153, 154]. The suppression of CXCR1/2 on neutrophils potentially protects the body from systemic inflammation favoring the development of liver failure. Inhibiting CXCR1/2 in a specific cell type could be a reasonable solution for detrimental hyper-inflammatory or immunosuppressive effects while maintaining the needed immune function and regenerative signaling. Furthermore, trials focused on the cell-specific CXCL8-CXCR2 anatomization still need to be studied in the near future (Table 4).

**Table 4:** List of CXCL8-CXCR2 inhibitors in clinical trials

Target	Drugs	Current status	Clinical Trials Identifier
**CXCL8**			
Antibodies	HuMax-IL8	Malignant solid tumor [157]	NCT02536469
**CXCR2**			
Small molecular inhibitors	Reparixin	IRI of liver transplant [158]; tumor [159]	NCT03031470; NCT02370238;
	Danirixin	COPD [160]; viral disease [161]	NCT03034967; NCT02469298

## Conclusion

Liver cells inevitably encounter and cope with MAMPs, PAMPs, and potentially harmful particles to protect the organism from infection or hyper-inflammation. During these processes, the CXCLs-CXCR2 axis is a crucial biological pathway that might be key for understanding liver injury during life-threatening infection. CXCR2 is also widely expressed in leukocytes and lymphocytes, controlling chemotaxis, inflammatory signaling, and mediating survival, proliferation, and repair during liver homeostasis, inflammation, and beyond. Inflammation and infection enhance CXCR2 expression on cells, especially neutrophils, where it activates pathogen clearance. In contrast, prolonged CXCR2 activation results in receptor desensitization and internalization. In sepsis, the frequently occurring initial cytokine storm can desensitize CXCR2 early, incapacitating immune cells to migrate to the side of infection efficiently and reducing the regenerative capacity of non-immune cells essential for liver regeneration. The close connection of CXCL8 and CXCR2 to sepsis onset and progression makes them biomarkers and therapeutic targets worth exploring. However, CXCR2 is involved cell-type specifically with multiple immunological and metabolic processes rendering one-target and one-time fits-all strategies unlikely to be a reliable solution to treat liver injuries, particularly during infection. Thus, finding checkpoints for balancing CXCR2 expression and function will be a crucial future goal in treating liver-related diseases.

## Supplementary Information

**Additional file 1.**

**Additional file 2.**

**Additional file 3.**

## Data Availability

The sequences, sequence annotations, and protocols used in Fig. 1 are available in Supplementary Information 1.
