# Supplementary material for: The immunological function of CXCR2 in the liver during sepsis
Source: J Inflamm (Lond). 2022 Nov 30;19:23. doi: 10.1186/s12950-022-00321-y (PMC9714102; doi:10.1186/s12950-022-00321-y)
Supplement: Supplementary file 1 — Additional file 1. [file 12950_2022_321_MOESM1_ESM.docx]

Supplementary Information

The immunological function of CXCR2 in the liver during sepsis.

Na Liu^1^, Michael Bauer^1,2^, Adrian T. Press^1,2,3^

^1^ Jena University Hospital, Department of Anesthesiology and Intensive Care Medicine, Friedrich-Schiller-University, Am Klinikum 1, 07747 Jena, Germany

^2^ Jena University Hospital, Center for Sepsis Control and Care, Am Klinikum 1, 07747 Jena, Germany

^3^ Friedrich-Schiller University Jena, Medical Faculty, Kastanienstr. 1, 07747 Jena

**Correspondence:** Jun.-Prof. Dr. Adrian T. Press, Jena University Hospital, Am Klinikum 1, 07747 Jena, Phone: +493641 9323139, eMail: adrian.press@med.uni-jena.de

# Content

[1. Generating the phyletic trees 2](#_Toc99708741)

[2. R-code 2](#_Toc99708742)

[3. Input sequence Data Figure 1a 2](#_Toc99708743)

[4. Input sequence Data Figure 1b 3](#_Toc99708744)

[5. References 3](#_Toc99708745)

# Generating the phyletic trees

Input sequence data are obtained from NCBI Gene Bank and Protein. [1] All sequence data were saved in the FASTA format. The Accession IDs of utilized sequences are provided below for both figures.

The data are analyzed and visualized using **R CRAN** (R version 4.1.2) [2] in combination with **RStudio** (free-distribution, version 2021.9.1.372) and the following R packages: **Biostrings** (version 2.62.0) [3], **ggtree** (version 3.2.1) [4], **treeio** (version 1.18.1) [5] and **ape** (version 5.6-2) [6], **ggplot2** (version 3.3.5) [7]. The Figures were ultimately formatted using **Inkscape™** (Version 1.1.2). [8]

# R-code

# Input sequence Data Figure 1a

**The input sequence data are obtained from NCBI Gene Bank. The accession numbers and respective gene symbols are provided below.**

- HsCXCR1: NP_000625.1
- HsCXCR2: XP_016859479.1
- HsCXCR3: NP_001495.1
- HsCXCR4: NP_001008540.1
- HsCXCR5: NP_001707.1
- HsCXCR6: NP_001373364.1
- MmCXCR1: XP_006496019.1
- MmCXCR2: XP_006495701.1
- MmCXCR3: NP_034040.1
- MmCXCR4: NP_001343438.1
- MmCXCR5: NP_031577.2
- MmCXCR6: NP_109637.3

# Input sequence Data Figure 1b

**The input sequence data for Figure 1b are obtained from NCBI Protein. The accession numbers and respective gene symbols are provided below.**

- Human CXCR1: P25024.2
- Rabbit CXCR1: P21109.2
- Rat CXCR1-like protein: P70612.1
- Gorilla CXCR1: P55919.1
- Chimpanzee CXCR1: P55920.1
- Human CXCR2: P25025.2
- Mouse CXCR2: P35343.1
- Rabbit CXCR2: P35344.1
- Rat CXCR2: P35407.2
- Gorilla CXCR2: Q28422.1
- Rhesus CXCR2: Q28519.1
- Chimpanzee CXCR2: Q28807.2
- Bovine CXCR2: Q28003.1

# References

1. Bethesda (MD): National Library of Medicine (US), N.C.f.B.I. *National Center for Biotechnology Information (NCBI)*. 1988 [cited 2021 01.04.2022]; Available from: <https://www.ncbi.nlm.nih.gov/>.

2. Team, R.C. *R: A language and environment for statistical computing. R Foundation for Statistical Computing*. 2017; Available from: <https://www.R-project.org/>.

3. H. Pagès, P.A., R. Gentleman, S. DebRoy. *Biostrings: Efficient manipulation of biological strings. R package version 2.62.0.*2021; Available from: <https://bioconductor.org/packages/Biostrings>.

4. Yu, G., *Using ggtree to Visualize Data on Tree-Like Structures.* Curr Protoc Bioinformatics, 2020. **69**(1): p. e96.

5. Wang, L.G., et al., *Treeio: An R Package for Phylogenetic Tree Input and Output with Richly Annotated and Associated Data.* Mol Biol Evol, 2020. **37**(2): p. 599-603.

6. Paradis, E. and K. Schliep, *ape 5.0: an environment for modern phylogenetics and evolutionary analyses in R.* Bioinformatics, 2019. **35**(3): p. 526-528.

7. Wickham, H., *ggplot2: Elegant Graphics for Data Analysis.* . 2009: Springer-Verlag New York,.

8. Yuan, S., et al., *PyMOL and Inkscape Bridge the Data and the Data Visualization.* Structure, 2016. **24**(12): p. 2041-2042.
